# Metabolome-Based Genome-Wide Association Study Provides Genetic Insights Into the Natural Variation of Foxtail Millet

**DOI:** 10.3389/fpls.2021.665530

**Published:** 2021-07-27

**Authors:** Wei Wei, Shuangdong Li, Yixiang Wang, Bin Wang, Guangyu Fan, Qisen Zeng, Fang Zhao, Congping Xu, Xiaolei Zhang, Tang Tang, Xiaolei Feng, Jian Shi, Gaolei Shi, Weiqin Zhang, Guoliang Song, Huan Li, Feng Wang, Yali Zhang, Xinru Li, Dequan Wang, Wenying Zhang, Jingjing Pei, Xiaoming Wang, Zhihai Zhao

**Affiliations:** ^1^Institute of Millet, Zhangjiakou Academy of Agricultural Science, Zhangjiakou, China; ^2^Wuhan Metware Biotechnology Co., Ltd., Wuhan, China

**Keywords:** foxtail millet, metabolic profiling, flavonoids, phenolamides, natural variation, mGWAS

## Abstract

The plant metabolome is considered as a bridge between the genome and the phenome and is essential for the interaction between plant growth and the plant environment. Here, we used the liquid chromatography-tandem mass spectrometry method to perform a widely targeted metabolomics analysis of 150 millet germplasm and simultaneous identification and quantification of 330 annotated metabolites. Comparing the metabolic content of different millets revealed significant natural variation of both primary and secondary metabolites, including flavonoids, phenolamides, hydroxycinnamoyl derivatives, nucleotides, and lipids, in the millets from India and the north and south of China; among them, some of the flavonoids are the most prominent. A total of 2.2 TB sequence data were obtained by sequencing 150 accessions of foxtail millet using the Illumina platform. Further digging into the genetic basis of metabolites by mGWAS analysis found that cyanidin 3-*O*-glucoside and quercetin *O*-acetylhexside are concentratedly located at 43.55 Mb on chromosome 5 and 26.9 Mb on chromosome 7, and two Lc were mined as candidate genes, respectively. However, the signals of luteolin 7-*O*-glucoside and kaempferol 3-*O*-glucoside were also detected at 14.36 Mb on chromosome 3, and five glycosyltransferase genes on this loci were deemed to regulate their content. Our work is the first research to use mGWAS in millet, and it paves the way for future dissection of complex physiological traits in millet.

## Introduction

As a healthy food material for the development of functional foods and natural antioxidant resources, foxtail millet (*Setaria italica*) is an important grain crop. It is characterized by adaptation to unfavorable ecological conditions, such as irregular and untimely rainfall, high-salinity soil, and high temperatures (Doust et al., 2009; Aidoo et al., 2016). The foxtail millet has been domesticated by humans from its wild species, green foxtail (*Setaria viridis*), in Northern China for over 8000 years (Barton et al., 2009), and it is widely cultivated as a dietary staple in the world, particularly in China and India with their rich and diverse germplasm (Bettinger et al., 2010; Lata et al., 2013). The metabolome is playing an increasingly important role in providing plants with environmental adaptability, Anthocyanins are commonly helped plants in response to different abiotic stresses, including drought, salinity, excess light, and extreme temperatures of hot and cold (Garriga et al., 2014; Kovinich et al., 2015; Miki et al., 2015). Investigation of the metabolome in rice and maize core collections has demonstrated significant natural variation of secondary metabolites, such as flavonoids, phenolamides, hydroxycinnamic acid derivatives, and terpenoids, in these species (Chen et al., 2014; Dong et al., 2014; Wen et al., 2014). For example, flavonoids, such as flavone mono-*C*-hexosides, malonylated flavone *O*-hexosides, and flavone 5-*O*-glycosides, accumulated in relevantly lower levels in the rice subspecies japonica than that in indica, while phenolamides, such as *N*-*p*-coumaroyl spermidine and *N*-sinapoyl agmatine/putrescine, accumulated at significantly higher levels in japonica than in indica (Dong et al., 2014; Wen et al., 2014). Among them, the natural variation of flavone *O*-glycosides has been demonstrated to determine the variation of UV tolerance in rice accessions (Peng et al., 2017). However, investigations of natural variation in metabolic traits, as well as their relationship with complex agronomic traits in foxtail millet are lacking.

In recent years, with the rapid development of gene chip and genome sequencing technology, high-quality genetic linkage maps and genome sequence information has been obtained in most crops. At the same time, there has been a continuous improvement in plant metabolite detection technology allowing more metabolites can be detected (Chen et al., 2013; Alvarez-Rivera et al., 2019; Ballesteros-Vivas et al., 2019). Combine these two aspects, produced the genome-wide association study, and metabolomics analysis (mGWAS) has become a powerful tool of forwarding genetics, which is suitable for exploring the genetic and biochemical basis of plant metabolism; thus far, it has been applied on metabolite structure analysis and functional genomics. Through mGWAS analysis of 805 metabolic traits in wheat, 1098 marker–metabolite associations were obtained, 26 candidate genes were unearthed, and the *in vitro* enzyme activity experiment revealed the main modification pathways of flavonoids in wheat (Chen et al., 2020). In the highland barley (Qingke), mGWAS was used to explain the accumulation of phenylpropane metabolic pathways in the high-level UV-B process of highland barley for its two aspects (being constitutive and inducible), and the genetic basis was studied (Zeng et al., 2020). In rice, the combined analysis of mGWAS and pGWAS proved that metabolic traits and phenotypes are interlinked, and new candidate genes for grain color and size were discovered. It provides a powerful strategy for interactive functional genomics and metabolomics in plants, especially the cloning of minor QTLs for complex phenotypic traits (Chen et al., 2014).

In this study, we collected a total of 150 diverse foxtail millet varieties from a majority of the tropical and temperate regions. We performed profiling of comprehensive metabolic and comparative analysis of metabolites at the primary and secondary metabolites in the millet population. Flavonoids, phenolamides, hydroxycinnamoyl derivatives, nucleotides, and lipids have an obvious natural variation of millet leaves in India and the north and south of China. Then, we used mGWAS to conduct preliminary genetic research on important flavonoid aglycone, identified two anthocyanin regulatory *Lc* proteins that affect the content of cyanidin 3-*O*-glucoside and quercetin *O*-acetylhexoside, five glycosyltransferase genes that regulate the synthesis of luteolin 7-*O*-glucoside and kaempferol 3-*O*-glucoside. The natural variation of metabolites may provide a theoretical basis for revealing that millet has different growth and development environments.

## Materials and Methods

### Plant Materials

The 150 millet varieties used in this study were taken from a collection of cultivated germplasm (Supplementary Table 1). The leaves at the five-leaf stage were collected from those varieties. The populations were evaluated in natural field conditions in the experimental farm of Zhangjiakou Academy of Agricultural Science, Zhangjiakou, China (144°88′N, 40°77′E), in a randomized complete block design. In each line was planted in a two-row plot of 1.5 m length with the spacing of 0.1 m between plants and 0.2 m between rows. Field management, including irrigation, fertilization, weeding, and pest control, followed the standard agricultural practices in millet production. Samples were taken from three different plants per line and pooled for each biological replicate. Two biological replicate leaf samples obtained from the same accessions were used for the GWAS. All the samples were harvested at 10:00–12:00 on that day, placed in liquid N2 immediately, and stored at −70°C until vacuum freeze-drying.

The 150 accessions used in this study were characterized by whole genome re-sequencing. DNA was isolated from young leaves and sequencing libraries with short inserts were constructed following the manufacturer’s instructions (Illumina). The samples were sequenced on an Illumina HiSeq 4000 platform. To retain reads of high quality, reads with fewer than 5% N (missing) bases and with fewer than 50% of bases of base quality <5 were deemed as cleaned reads. All other reads were discarded.

### Chemicals

All the chemicals were of analytical reagent grade. Gradient-grade methanol, acetonitrile, and acetic acid were purchased from Merck Company, Germany.^1^ The water was doubly deionized with a Milli-Q water purification system (Millipore, Bedford, MA, United States). Authentic standards were purchased from ANPEL, Shanghai, China,^2^ BioBioPha Co., Ltd.,^3^ and Sigma-Aldrich, United States.^4^ Standards stock solutions were prepared using water, methanol, and/or dimethyl sulfoxide (DMSO) as the solvent and stored at −20°C. Combined standard solutions of chemicals were prepared just before use by mixing individual stock solutions and diluting these mixtures with 70% aqueous methanol.

### Sample Preparation and Extraction

The freeze-dried leaves were crushed using a mixer mill (MM 400, RETSCH) with zirconia beads for 1.5 min at 30 Hz. A 100 mg mass of powder was weighed and extracted overnight at 4°C with 1.0 ml of 70% aqueous methanol. Following centrifugation at 10,000 × *g* for 10 min, the extracts were filtered (SCAA-104, 0.22 μm pore size; ANPEL, Shanghai, China^5^) before LC-MS analysis.

### LC-MS Conditions

The sample extracts were analyzed using an LC-ESI-MS/MS system (HPLC, Shim-pack UFLC SHIMADZU CBM30A system^6^; MS, Applied Biosystems 6500 Q TRAP^7^). The analytical conditions were as follows, HPLC: column, Waters ACQUITY UPLC HSS T3 C18 (1.8 μm, 2.1 mm × 100 mm); solvent system, water (0.04% acetic acid): acetonitrile (0.04% acetic acid); gradient program, 100:0 V/V at 0 min, 5:95V/V at 10.0 min, 5:95V/V at 11.0 min, 95:5 V/V at 11.1 min, 95:5 V/V at 15.0 min; flow rate, 0.35 ml/min; temperature, 40°C; and injection volume: 5 μl. The effluent was alternatively connected to an ESI-triple quadrupole-linear ion trap (Q TRAP)-MS.

LIT and triple quadrupole (QQQ) scans were acquired on a triple quadrupole-linear ion trap mass spectrometer (Q TRAP) using an API 6500 Q TRAP LC/MS/MS System, which was equipped with an ESI Turbo Ion-Spray interface operated in a positive ion mode and controlled by Analyst 1.6.3 software (AB Sciex). The ESI source operation parameters were as follows: ion source, turbo spray; source temperature 550°C; ion spray voltage (IS) 5500 V; ion source gas I (GSI), gas II (GSII), and curtain gas (CUR) were set at 55, 60, and 30.0 psi, respectively; and the collision gas (CAD) was high. Instrument tuning and mass calibration were performed with 10 and 100 μmol/L polypropylene glycol solutions in QQQ and LIT modes, respectively. The QQQ scans were acquired as MRM experiments with the collision gas (nitrogen) set to 5 psi. The DP and CE for individual MRM transitions were performed with further DP and CE optimization. A specific set of MRM transitions was monitored for each period according to the metabolites that were eluted within this period.

Obtaining the highly reproducible metabolite signals with the production spectra. Based on the annotation, commercially available standards were purchased and analyzed using the same profiling procedure as the extracts.

### Genome-Wide Association Analyses

Only SNPs with minor allele frequencies R 0.05 and with the number of varieties having the minor allele in a (sub) population R6 were used to carry out GWAS. After obtaining the metabolome data, combined with the genotypes of 150 related populations, mGWAS analysis was first performed on the related population data. GWAS analysis used the compressed mixed linear model (cMLM), which integrates metabolic phenotype, genotype, population structure, and genetic relationship, to analyze, obtaining the association with each genotype (SNP) and phenotype *P*-value. After Bonferroni correction (*P* = 0.05/1,963,697), it was used to correct the threshold of localization, where the significant threshold of mGWAS after correction is *P* ≤ 1.8 × 10^–6^, and thus to locate the area significantly associated with the metabolic phenotype. Then, we used R to draw Manhattan graph and Q–Q graph in order to visually show the significant associated area and model effect.

### Statistical Analysis

Principal component analysis (PCA) was performed with log2 transformed metabolite data to improve the normality. For hierarchical clustering analysis (HCA) in the study of developmentally controlled accumulation and natural variation of metabolites, metabolite data were firstly log2 transformed, followed by a min–max normalization. To construct the neighbor-joining tree, the data matrix was generated from 150 millet varieties and 1335 detected metabolites, which represented the contents of each metabolite in different populations. The pairwise population distance was used to construct a neighbor-joining tree using the software PHYLIP (version 3.69). The software TreeView and MEGA7 were used for visualizing the phylogenetic tree. For HCA, the “heatmap.2” function in the “gplot” R-package was utilized to generate the heatmap, and various R programming tools were used to plot data.^8^ Identification of differential accumulation of metabolites between different tissue/varieties was determined by partial least squares-discriminate analysis (PLS-DA) with the VIP values (Variable Importance for the Projection) ≥0.8, followed by both ANOVA (*p* ≤ 0.05) and fold-change (≥1.5 or ≤0.67). PCA and PLS-DA were performed with SIMCA-P version 14.0.

### *F*_ST_ Analysis

Developed as a special case of Wright’s *F* statistics, the fixation index (*F*_ST_) is one of the most commonly used statistics in population genetics, and it is a measure of population differentiation due to genetic structure, which is frequently estimated from genetic polymorphism data, such as SNPs (Holsinger and Weir, 2009). Estimates of *F*_ST_ can identify regions of the genome that have been the target of selection, and comparisons of *F*_ST_ from different parts of the genome can provide insights into the demographic history of populations. For selection signature analysis, SNPs of the whole genome were used, and a sliding window approach (100-kb windows sliding in 10-kb steps) was employed to quantify genetic differentiation (*F*_ST_) for Indian and Northern Chinese accessions using the VCFtools software. Windows with *F*_ST_ values exceeding an empirical *F*_ST_ cutoff (top 5%) were regarded as highly differentiated regions. Windows separated by less than 200 kb were merged into a single non-overlapping region.

### Phylogenetic Analysis

The amino acid sequences of reported genes including *Lc* transcription factors and glycosyltransferase were obtained from NCBI according to their accession numbers.^9^ Candidate gene information in this study was obtained from the draft assembly of the millet genome (Bennetzen et al., 2012). The alignment of amino acid sequences was performed using ClustalW bundled in MEGA 5, and neighbor-joining trees were constructed using MEGA 5 software with all default parameters. The reliability of the reconstructed tree was evaluated using a bootstrap test with 1000 replicates.

### qRT-PCR Analysis

To validate the RNA-seq output, seven candidate genes were selected and analyzed by qRT-PCR. The total RNA was extracted from selected samples using 1.5 × CTAB buffer. The qRT-PCR was performed on a 7500 Fast Real-Time PCR System (AB Ltd.) using the SuperReal PreMix Plus (SYBR Green) kit (Tiangen Biotech Co., Ltd., Beijing, China) according to the manufacturer’s instructions. The PCR was executed under the following process: 95°C for 5 min for pre-degeneration, then 35 cycles of 94°C for 30 s, 56°C for 30 s, and 72°C for 90 s. Each experiment was performed in triplicate for biological and technical repeats. The sequences of primers for qRT-PCR are listed in Supplementary Table 13.

## Results

### Metabolic Characterization of Foxtail Millet

To obtain a global view of the metabolic variation in foxtail millet cultivars, we performed metabolic profiling analyses with leaves at the five-leaf stage from 150 accessions using a previously reported broadly targeted liquid chromatography-tandem mass spectrometry (LC-MS/MS)-based metabolic profiling method (Chen et al., 2013; Supplementary Table 1). By constructing a millet leaf MS^2^ spectral tag (MS^2^T) library, we finally obtained the 1335 highly reproducible metabolite signals with the production spectra (Supplementary Tables 2, 3). Specifically, 133 were identified with the commercial standards, and 197 were putatively annotated as previously described (Dong et al., 2014; Wang et al., 2016; Li et al., 2018). Among which, we identified 155 primary metabolites, including 31 amino acids with their derivatives, 24 nucleotides and their derivatives, 5 carbohydrates, 23 organic acids, and 72 lipids, and a number of secondary metabolites, including 42 flavone *O*-glycosides, 22 flavone *C*-glycosides, 13 flavonol *O*-glycosides, 9 flavanones, 6 anthocyanins, 6 flavonolignans, 12 hydroxycinnamic acid derivatives, 11 hydroxycinnamoyl quinates, 15 phenolamides, 5 phytohormones, 14 benzoic acid derivatives, 4 coumarins, 3 alkaloids, 4 vitamins, and 9 others (Supplementary Figure 1A).

### Natural Variation of the Metabolome in Foxtail Millet

**FIGURE 1:**
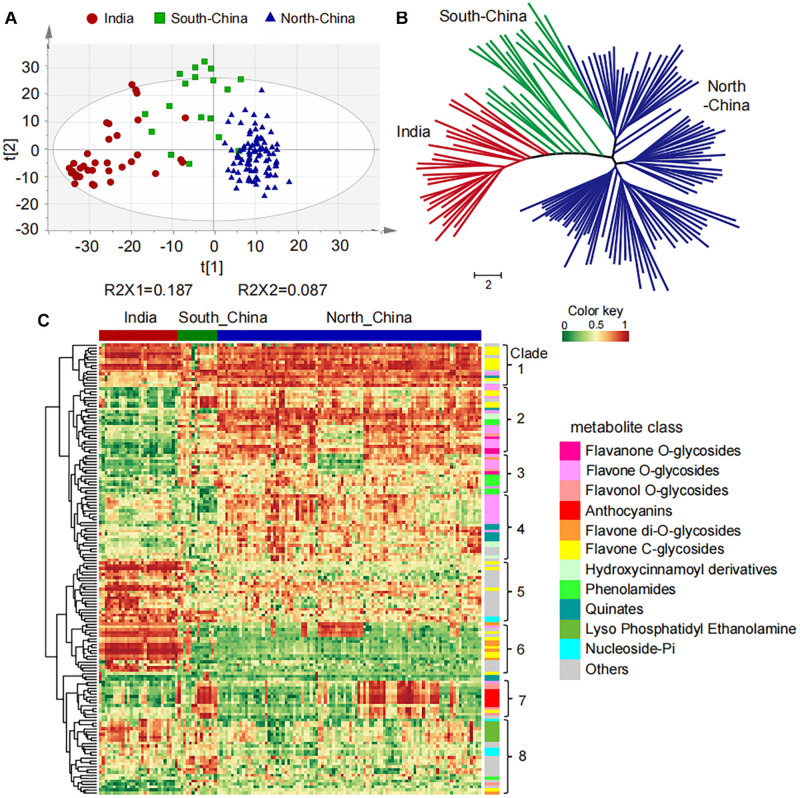
Natural variation and population structure of 150 millet accessions based on metabolic profiling. **(A)** Score plot of principal component analysis (PCA) for datasets of millet leaf metabolites. The red, green, and blue dots represent samples from India, the south and north of China, respectively. **(B)** Neighbor-jointing phylogenetic tree of 150 millet accessions with leaf metabolites. Three major clades, represented by India (clade I), South-China (clade II), and North-China (clade III), were identified from the tree. The scale bar indicates the simple matching distance. **(C)** Heatmap visualization of relative differences of metabolites that showed significant differential accumulation among Indian and Chinese millet accessions. Each millet accession is visualized in a single column and each metabolite is represented by a single row. Data of the content value of each metabolite were normalized to complete linkage hierarchical clustering. Red indicates high abundance, and green indicates low relative metabolic contents.

To study the natural variation of metabolites among the millet varieties, high-throughput quantification of the 1335 metabolic features was then carried out in the leaf from these varieties for widely targeted metabolic analysis. More than 50% of the metabolites showed coefficients of variation (CV) greater than 50% (Supplementary Figure 1B), suggesting their significant variation in millets. Further, the PCA separated the 150 millet accessions into three groups clearly, and a deep insight into the geographical distribution of them revealed that they were mainly represented by the millet varieties from India, the north of China, and the south of China (Figure 1A). The clustering analysis based on the levels of metabolites yielded a similar result and divided these varieties into three major clades from the neighbor-joining tree (Figure 1B), indicating significant metabolic variations in foxtail millets from different geographical locations. When comparing the metabolic accumulation in different groups of millet, fold-change (FC ≥1.5 or ≤0.67), VIP (VIP ≥ 0.8), and *P*-value (*p* < 0.05) were used to evaluation of the metabolite contents and 617 metabolites showed significant variations among the millet varieties (Supplementary Table 4), among which 155 metabolites were annotated. Visualization of the metabolic profile in the millet population by hierarchical cluster analysis revealed a clear phenotypic variation in terms of their abundance among the millets from India, the south and north of China, and the 155 metabolites could be clearly grouped into two main clusters with eight subclusters (Figure 1C). Overall, metabolites in clade 1, clade 2, clade 3, clade 4, and clade 7 showed significantly higher levels in millet from the south and/or north of China than that in India, and they were mainly represented by flavone *C*-glycosides, flavone *O*-glycosides, flavonol *O*-glycosides, anthocyanins, hydroxycinnamic acid derivatives, and phenolamides. The metabolites in clade 5, clade 6, and clade 8, however, displayed lower levels in millet from the south and/or north of China when compared to the millet in India, and were mainly represented by *C*-glycosyl-flavone *O*-glycosides, flavone di-*O*-glycosides, nucleoside phosphate, and glycerophospholipids (Figure 1C), disclosing significant natural variation in both primary and secondary metabolites in millets from different areas. These observations suggested that flavonoid *C*/*O*-glycosides, phenolamides and glycerophospholipids might function as important phytochemical protectants for millets cultivated in different areas.

**FIGURE 2:**
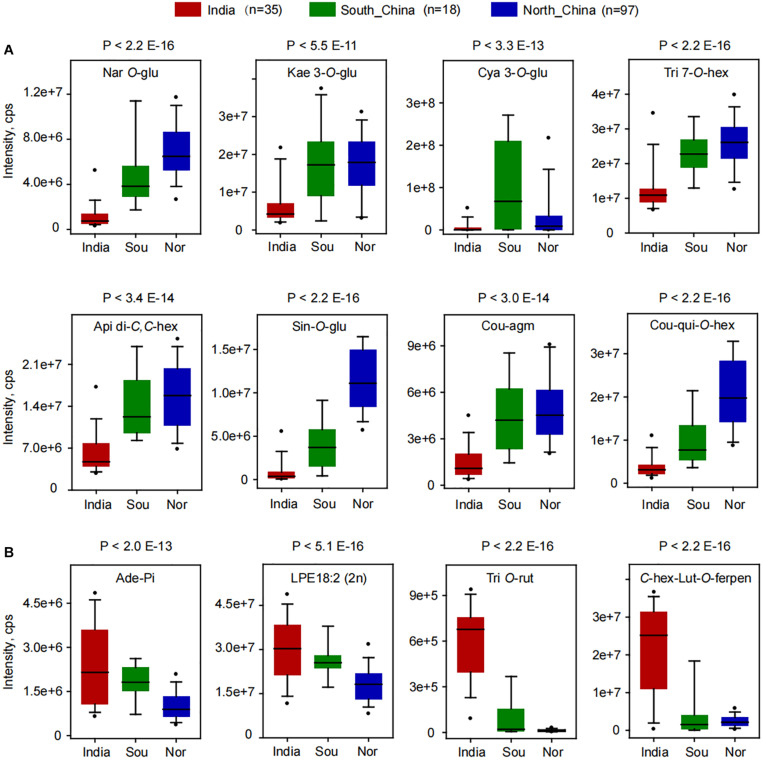
Boxplot for the content of differentially accumulated metabolites among the millets from India, the south of China (Sou), and the north of China (Nor). **(A)** Significantly higher metabolites were found in millet from northern China. **(B)** Significantly higher metabolites were found in millet from India. Ade-Pi, adenosine 5′-monophosphate; Api di-*C*,*C*-hex, apigenin di-*C*,*C*-hexoside; *C*-hex-Lut-*O*-ferpen, *C*-hexosyl-luteolin *O*-feruloylpentoside; Cou-agm, *N*′-*p*-coumaroyl agmatine; Cou-qui-*O*-hex, 3-*O*-*p*-coumaroyl quinic acid *O*-hexoside; Cya 3-*O*-glu, cyanidin 3-*O*-glucoside (Kuromanin); Kae 3-*O*-glu, kaempferol 3-*O*-glucoside (Astragalin); LPE18:2 (2n), LysoPE 18:2 (2n isomer); Nar *O*-glu, naringenin *O*-glucoside; Sin-*O*-glu, 1-*O*-beta-D-glucopyranosyl sinapate; Tri 7-*O*-hex, tricin 7-*O*-hexoside; Tri *O*-rut, tricin *O*-rutinoside.

A deep insight into the differentially accumulated metabolites revealed that, compared to Indian millet, 62 metabolites, including 18 flavone *O*-glycosides, 6 flavone *C*-glycosides, 5 flavanones, 5 flavonols, 6 anthocyanins, 6 hydroxycinnamoyl acid derivatives, 7 phenolamides, and 6 hydroxycinnamoyl quinates, were shown to be significantly higher in millet from the north of China, while 45 metabolites, including 7 flavone *O*-glycosides, 9 flavone *C*-glycosides, 4 flavanone, 7 nucleotides, and 6 glycerophospholipids, were shown to have significantly lower levels (Figure 1C and Supplementary Table 4). Among which, flavonoid *O*-glycosides belonged to different classes, such as naringenin *O*-glucoside (GZ0896), kaempferol 3-*O*-glucoside (GZ2519), cyanidin 3-*O*-glucoside (GZ2192), and tricin 7-*O*-hexoside (GZ2643) displayed 5.9, 1.8, 5.0, and 2.0 times over-accumulation in millet from north of China than that from India, respectively (Figure 2A). Flavone *C*-glycoside, hydroxycinnamic acid derivatives, phenolamides, and hydroxycinnamoyl quinates, such as apigenin di-*C*,*C*-hexoside (GZ2331), 1-*O*-beta-D-glucopyranosyl sinapate (GZ0627), *N*′-*p*-coumaroyl agmatine (GZ2195), and 3-*O*-*p*-coumaroyl quinic acid *O*-hexoside (GZ0446), also showed significantly higher levels in millet from the north of China with average levels up to 2.5, 12.0, 3.3, and 5.8-fold, respectively (Figure 2A). On the contrary, there were significantly lower levels of flavone di-*O*-glycoside and *C*-glycosyl-flavone *O*-glycosides, represented by tricin *O*-rutinoside (GZ2860) and *C*-hexosyl-luteolin *O*-feruloylpentoside (GZ2521), and nucleoside phosphate and glycerophospholipids, such as adenosine 5′-monophosphate (GZ1824) and LysoPE 18:2 (GZ3304), in millet from the north of China than that in India, with average decreased levels up to 0.4, 0.6, 0.02, and 0.1-fold (Figure 2B), respectively. A similar result could be observed when comparing the metabolic variation between the millet from India and the south of China. A total of 57 metabolites showed significantly higher, and 46 metabolites showed significantly lower levels in millet from the south of China compared to Indian millet (Figure 2 and Supplementary Table 4).

### Genetic Basis of Metabolic Differences in Foxtail Millet

**FIGURE 3:**
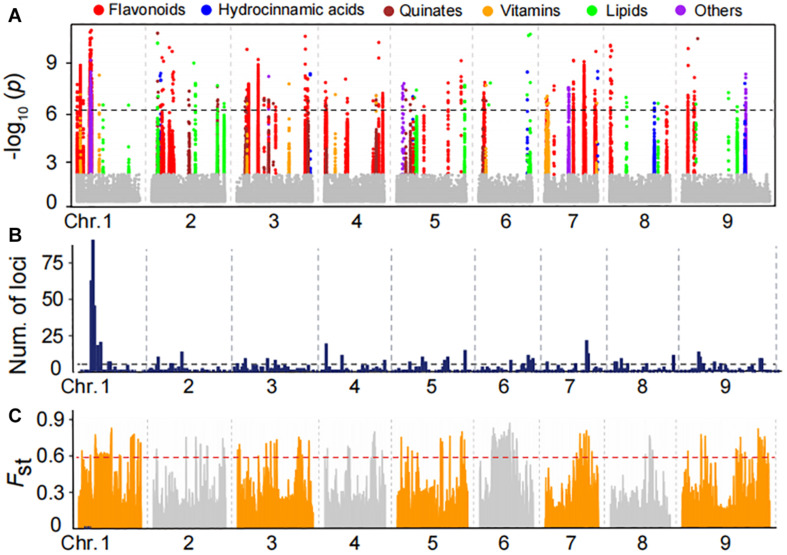
Manhattan plots of mGWAS results and genetic differentiation of mGWAS loci between Indian and Chinese millet. **(A)** Manhattan plots of mGWAS results with the genetic association for the leaf metabolic features in millet. The strength of association for the metabolites is indicated as the negative logarithm of the *p*-value for the LMM model. All metabolite-SNP associations with *P*-values below 1.8E–06 are plotted against genome location in intervals of 1 Mb. **(B)** Distribution of mGWAS signals across the millet genome. **(C)** Distribution of differentiation (*F*_ST_ values) from the comparison of the Indian and northern Chinese millet populations. The regions above the dashed line in the *F*_ST_ values distribution are in the 5% right tail of the empirical distribution (*F*_ST_ is 0.59).

To uncover the genetic control of natural variation in metabolites, the 150 foxtail millet accessions of mini-core collection of the varieties were subjected to Illumina Hiseq 4000 platform and generated approximately 2.2 TB of clean data. Pair-end reads were mapped against the reference genome of millet genome reference (Bennetzen et al., 2012) and a final set of 1,963,697 high-quality SNPs were identified by selecting SNPs with missing rates of less than 10%. We then performed mGWAS for the foxtail millet population using these SNPs with a cMLM that results in avoiding fitting transition and saves calculation time. Thereby, we generated a total of 973 lead SNPs for 157 metabolites (Supplementary Table 5), corresponding to 237 quantitative trait loci (Table 1 and Supplementary Tables 6, 7), underlying the genome-wide significant threshold *P*_cMLM_ = 1.8E−06 after Bonferroni correction. Statistical analysis showed 11.76% of the metabolites detected had at least one significant association, with an average of 6.2 associations per metabolite (Supplementary Table 7). The significant association for metabolites in different categories was illustrated (Figure 3A), and there were 47 potential mGWAS hot spots, which were mainly located in chromosome 1, 5, and 9, respectively (Figure 3B and Supplementary Table 9). Moreover, we identified that a wide range of metabolites within the same and different categories was co-localized by mGWAS. For example, we observed flavonoids in different subgroups, including anthocyanins, flavonol *O*-glycosides, and malonylated flavonol *O*-glycosides, comapped to 43.55 Mb on chromosome 5 and 26.9 Mb on chromosome 7. Chrysoeriol *C*-hexoside-*O*-feruloyl-*O*-pentoside (GZ2812) is an example of a metabolite co-mapping with metabolites in different classes; it comapped with 1-*O*-*p*-coumaroyl quinic acid (GZ0548) and 3-*O*-*p*-coumaroyl quinic acid *O*-hexoside (GZ0446), primarily at 9.3 Mb on chromosome 9 (Supplementary Table 9). These observations implied common genetic regulation of metabolites, mainly phenylpropanoids, in millet.

**TABLE 1:** Summary of genome-wide significant associations identified in the mGWAS.

Item	Population
Number of traits	157
Number of lead SNPs	971
Number of loci	237
SNPs above 20% of variation	821
Maximum explained variation (%)	60.3
Explained variation per SNP (%)	26.0

To identify the potential Indian and Chinese-differentiated signals at the whole-genome level, the *F*_ST_ value was calculated. Further scanning the regions by comparing Indian and Northern Chinese accessions identified genomic differential regions totaling 33.64 Mb in length, mainly distributed on chromosomes 1, 3, 6, 7, and 9 (Figure 3C and Supplementary Table 10). When comparing the significant mGWAS loci for the metabolites with the regions, we found that a total of 35 significant loci corresponding to 58 metabolites were overlapped in the Indian–Chinese differential regions (Supplementary Table 11). For example, the mGWAS had significant loci at 43.5 Mb on chromosome 5 for anthocyanis, and flavonol *O*-glycosides were located at the differentiated regions. These observations further revealed that the phenylpropanoid pathway was specifically selected during both natural selection and artificial domestication of millet.

### Functional Interpretation of GWAS for Anthocyanins and Flavonols

**FIGURE 4:**
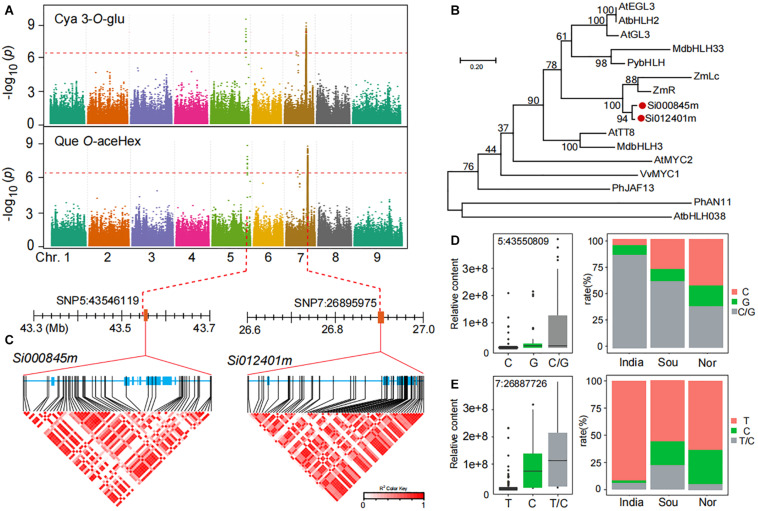
Genome-wide association study of anthocyanins and flavonols in millet. **(A)** Manhattan plots of mGWAS results of cyanidin 3-*O*-glucoside (Cya 3-*O*-Glu) and quercetin *O*-acetylhexoside (Que *O*-aceHex) in millet. The strength of association for the metabolites is indicated as the negative logarithm of the *p*-value for the LMM model. All metabolite-SNP associations with *p*-values below 1.8E–06 are plotted against genome location in intervals of 1 Mb. **(B)** An unrooted phylogenetic tree of the MYC protein was constructed as described in Methods. Bootstrap values >70% (based on 1000 replications) are indicated at each node (bar: 0.2 amino acid substitutions per site). **(C)** A representation of pairwise *r*^2^ values (a measure of LD) among all polymorphic sites in *Si000845m* and *Si012401m*, where the color of each box corresponds to the *r*^2^ value according to the legend. **(D,E)** Natural variation of nucleotide polymorphisms identified in the sequences of *Si000845m* and *Si012401m* and their allelic frequencies in millet from India, south of China (Sou), and north of China (Nor).

In our results, we found that the accumulation of anthocyanins, such as cyanidin 3-*O*-glucoside (GZ2192), and flavonol *O*-glycosides, such as quercetin *O*-acetylhexoside (GZ0824, precursor substance for anthocyanin synthesis), in Chinese millet is much higher than that of Indian millet according to GWAS analysis, and we found important identical loci related to their accumulation level (Figure 4A). A closer look revealed that the leading SNP 5:43546119 on chromosome 5 was significantly associated with the level of cyanidin 3-*O*-glucoside (*P*-value of 3.0E−09) and quercetin *O*-acetylhexoside (*P*-value of 1.1E−09), similarly, another leading SNP 7:26896246 on chromosome 7 was significantly associated with the level of cyanidin 3-*O*-glucoside (*P*-value of 5.7E−09) and quercetin *O*-acetylhexoside (*P*-value of 1.0E−09). When searching for the candidate genes in the locus on chromosome 5, we found that the *Si000845m* gene located 0.13 kb downstream of lead SNP 5:43546119 and was annotated as an anthocyanin regulatory *Lc* protein. Furthermore, when focused on another locus at chromosome 7, we found the gene *Si012401m* located ±4.0 kb upstream of lead SNP 7:26896246 and is also annotated anthocyanin regulatory *Lc* protein (Figure 4A). In a phylogenetic tree of reported MYC transcription factor, *Si000845m* and *Si012401m* were tightly clustered in one subclade with a gene, *ZmLc* (Figure 4B), reported to be involved in responding to low-temperature induction and regulating anthocyanin biosynthesis (Ludwig et al., 1989; Nathalie et al., 2000).

In searching for possible functional polymorphism(s) underlying the natural variation of anthocyanins and flavonol *O*-glycosides, we found a number of highly significant associations between non-synonymous SNPs in *Si000845m* and *Si012401m* and the levels of cyanidin 3-*O*-glucoside (Figure 4C). Among which, one allelic mutation (SNP 5:43550809) in the *Si000845m* coding region was significantly associated with levels of anthocyanins and flavonol *O*-glycosides, such as cyanidin 3-*O*-glucoside (Supplementary Table 12). This mutation resulted in significant polarity changes of amino acids between two groups (Allele I and II; Supplementary Table 12). The mean cyanidin 3-*O*-glucoside content in allele II (G) was significantly higher when compared with that in allele I (C), with *p*-values 2.5E−03 (Figure 4D and Supplementary Table 12). This is consistent with the significant correlation value between distribution differences in millet fields and two group ratios (Figure 4D). Similarly, a significant relationship (*p*-value of 1.0E−10) was observed between cyanidin 3-*O*-glucoside content and SNP7:26887726, which caused non-synonymous mutations and in *Si012401m* coding region (Figure 4E and Supplementary Table 12). Furthermore, there was a significant correlation between the two alleles ratio and the cyanidin 3-*O*-glucoside content of millet in different regions (Figure 4E).

Together, we assigned *Si000845m* and *Si012401m* as the candidate MYC transcription factors controlling the biosynthesis of both anthocyanins and flavonol *O*-glycosides, and we propose that genetic variants within their coding regions might contribute to the natural variation in levels of anthocyanins and flavonol *O*-glycosides in millet. We verified the expression levels of candidate genes in leaves of different genotype materials by using qRT-PCR and found that the expression level of *Si012401m* has the same trend as the content of cyanidin 3-*O*-glucoside (GZ2192) (Supplementary Figures 2A,B).

### Functional Interpretation of GWAS for Flavone *O*-Glycosides

**FIGURE 5:**
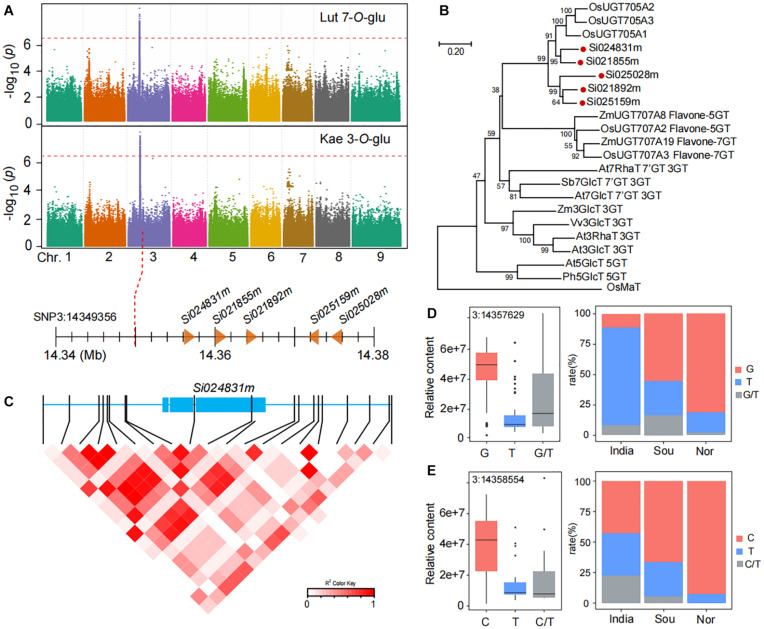
Genome-wide association study of flavone O-glycosides in millet. **(A)** Manhattan plots of mGWAS results of luteolin 7-*O*-glucoside (Lut 7-*O*-Glu), and kaempferol 3-*O*-glucoside (Kae 3-*O*-Glu) in millet. The strength of association for the metabolites is indicated as the negative logarithm of the *p*-value for the LMM model. All metabolite-SNP associations with *p*-values below 1.8E–06 are plotted against genome location in intervals of 1 Mb. **(B)** An unrooted phylogenetic tree of the UDP-glycosyltransferase was constructed as described in Methods. Bootstrap values >70% (based on 1000 replications) are indicated at each node (bar: 0.2 amino acid substitutions per site). **(C)** A representation of pairwise *r*^2^ values (a measure of LD) among all polymorphic sites in *Si024831m*, where the color of each box corresponds to the *r*^2^ value according to the legend. **(D,E)** Natural variation of nucleotide polymorphisms identified in the sequences of *Si024831m* and their allelic frequencies in millet from India, south of China (Sou), and north of China (Nor).

The accumulation of flavone-*O*-glycosides such as luteolin 7-*O*-glucoside (GZ0794) and kaempferol 3-*O*-glucoside (GZ2519) in millet from China was markedly higher than that in India. We found that two flavone-*O*-glycosides (luteolin 7-*O*-glucoside and kaempferol 3-*O*-glucoside) were identified at the same loci based on mGWAS (Figure 5A). The leading SNP3:14349356 on chromosome 3 is significantly associated with the level of luteolin 7-*O*-glucoside (*p*-value of 3.0E−8) and kaempferol 3-*O*-glucoside (*p*-value of 9.1E−10). We next mined candidate genes in the loci on chromosome 3, and we found five genes downstream of lead SNP3:14349356 and annotated them as related functions of glycosyltransferase: *Si024831m* (7.03 kb downstream of SNP3:14349356), *Si021855m* (10.77 kb downstream of SNP3:14349356), *Si021892m* (14.54 kb downstream of SNP3:14349356), *Si025159m* (21.98 kb downstream of SNP3:14349356), and *Si025028m* (25.25 kb downstream of SNP3:14349356). Phylogenetic analysis showed that five candidate genes have similar sequences to the genes in rice that confer flavone-*O*-glucosyltransferase (Figure 5B; Peng et al., 2017).

To investigate function variations of candidate genes, we analyzed all SNPs in the full length of the *Si024831m*. There are 20 SNPs associated with kaempferol 3-*O*-glucoside content (Figure 5C). Among them, SNP3:14357629 in *Si024831m* coding region and caused the content of luteolin 7-*O*-glucoside in millet of the G genotype group to be significantly higher than that of the T genotype group with a *p*-value of 7.23E−18 (Figure 5D and Supplementary Table 12). Indeed, non-synonymous SNP3:14358554 are also located in the coding region of the *Si024831m*, which makes the content of kaempferol 3-*O*-glucoside in different genotypes of millet differ significantly, with a *p*-value of 1.69E−4 (Figure 5E and Supplementary Table 12). Following this, we further analyzed the allele frequencies of three groups of millet from India, the north of China, and the south of China, and we found, that compared with India, millet from the two regions of China has a higher frequency of alleles with kaempferol 3-*O*-glucoside (C) and luteolin 7-*O*-glucoside (G) content and thus has a higher kaempferol 3-*O*-glucoside and luteolin 7-*O*-glucoside content (Figures 5D,E).

The expression level of *Si024831m* is highly correlated with the content of luteolin 7-*O*-glucoside (GZ0794), and its expression level in materials with high content is significantly higher than that in materials with low content (*p*-value = 9.65E−4) (Supplementary Figures 2C,D). However, there is no difference in the expression level of *Si021892m* between high and low kaempferol 3-*O*-glucoside (GZ2519) content materials (*p*-value = 0.32) (Supplementary Figures 2E,F).

## Discussion

### Identification and Natural Variation of Metabolites in Millet

Metabolites can not only reflect plant physiological states; they also play an important role in plant growth and the interaction between plant and environment (Keurentjes, 2009; Treutter, 2009; Saito and Matsuda, 2010; Zhang et al., 2020). Here, 1335 metabolites, including 330 annotated metabolites and 1005 unknown metabolites, were identified and quantified in leaves at the five-leaf stage from 150 accessions. We found 155 annotated metabolites were differentially enriched of millet in three regions of India, the south and north of China, among which were mainly glycosylated forms of flavonoids and Aromatic acylated metabolites. Based on our study, glycosylated forms of flavonoids in the millets were mainly represented by flavone/flavanone mono *C*-hexosides, flavone di-*C*,*C*-hexosides, *C*-hexosyl flavone-*O*-hexosides, flavone/flavanone/flavonol *O*-glycosides, flavone *O*-rutinosides, and anthocyanidin *O*-glycosides, coinciding with previous reports in rice and wheat (Moheb et al., 2011; Dong et al., 2014). Glycosylation modification of secondary metabolites is common in plants and has a wide range of important functions, mainly to respond to abiotic stress and help plants to complete recovery; enhance the antioxidant capacity of plants; and ensure the detoxification of harmful compounds in plants (Julien et al., 2016). Aromatic acylated metabolites detected were mainly represented by hydroxycinnamic acids and their *O*-aglycones, phenolamides, hydroxycinnamoyl quinates/shikimates, and *C*-hexosyl flavone-*O*-hydroxycinnamoyl hexosides. It is well known that the decoration of phytochemicals, such as glycosylation, acylation, methylation, hydroxylation, and prenylation, not only contributes to the diversity of metabolites in plants but is also essential for plant growth and adaptation to the environment (Fang et al., 2018; Wang et al., 2019).

A particular planting environment may cause a specific accumulation of related metabolites in subspecies. The significant natural variation of these decorated phytochemicals especially flavonoids and phenolamides in leaves could be found between the millets from the north of China and India. This difference is usually caused by plants adapting to different geographical environments, thus, combined with the characteristics of the geographical environment, studying the natural variation of metabolites can help us further understand their functions (Wen et al., 2014). Flavonoid aglycones in rice leaf, including flavone mono-*C*-hexosides, flavone *O*-glycosides, and malonylated flavone *O*-hexosides, and phenolamides, including *p*-coumaroyl agmatine/putrescine, showed significantly lower levels in *japonica* cultivated in the relative higher latitudes (represented by the north of China) than that in *indica* in the relative lower latitudes (represented by India and the south of China), these metabolites, however, displayed significantly higher levels in the millet from the north of China than that in India, which is important for UV-B protection in plants (Dong et al., 2014, 2015; Peng et al., 2017). In addition to the UV intensity, the temperature in different latitudes also has obvious differences. The low-temperature environment in high latitudes may cause the active oxygen produced, which leads to frost damage to the crops. As antioxidants, flavonoid aglycone and phenolamide can effectively help crops eliminate freezing damage (Schulz et al., 2015, 2016; Jian et al., 2020; Wong et al., 2020). This may be the reason for the specific enrichment of flavonoid aglycone and phenolamide in northern China. However, the exact mechanism underlying the different patterns of metabolic accumulation in rice and millet requires further investigation.

### Natural Variation of Metabolites in Millet Can Be Used in Biomarker

Metabolites can also be used as biomarkers for subspecies in different planting environments. It has been suggested that flavone *C*-pentosides/hexosides, and malonylated flavonoid *O*-glycosides, are good candidate metabolic markers for distinguishing characteristics of indica and japonica rice varieties (Chen et al., 2013; Dong et al., 2015). Based on the significantly huge variation of metabolites in millet, the differentially accumulated metabolites, such as flavonoids, phenolamides, and hydroxycinnamoyl quinates, could be used as biomarkers for the discrimination of millet from different areas. For instance, the anthocyanins pelargonidin-3-*O*-malonyl-malonylhexoside (GZ2423) has obvious differences in the millet leaves of the three regions. Among them, the content of millet leaves in southern China is the highest, which is 596.75 times and 15.48 times that in India and northern China, respectively. Not only that, the content of it in millet leaves in northern China and India is also significantly different, with a multiple of 38.54 (Supplementary Table 4). Hence, Pelargonidin-3-*O*-malonyl-malonylhexoside can help us determine the source of millet materials. In this study, flavonoid aglycone has obvious differences in millet leaves in different regions and can be used as a marker metabolite to guide planting and breeding.

### Homologous Genes of Candidate Genes Are Functionally Conserved in Other Plants

As a kind of anthocyanin, cyanidin 3-*O*-glucoside can effectively enhance the tolerance of plants in drought, cold, and high-light environments (Becker et al., 2013; Kovinich et al., 2015). It is formed from dihydroquercetin, catalyzed, and modified by dihydroflavonol 4-reductase (DFR) and anthocyanin synthase (ANS) UDP-glycosyltransferase (UGT) in plants (Nesi et al., 2000; Baudry et al., 2004). In this study, for cyanidin 3-*O*-glucoside and quercetin *O*-acetylhexoside, we locate two candidate genes *Si000845m* and *Si012401m* on chromosomes 5 and 7, respectively, and their functional annotated are both anthocyanin regulatory *Lc* protein. Comparative genomics shows that homologous genes from a common ancestor usually have conserved functions, which provides a basis for us to confirm the function of candidate genes in millet (Devos, 2005; Murat et al., 2017; Wang et al., 2019). Two candidate genes are highly homologous to the anthocyanin regulatory *Lc* protein in maize (Figure 4B), and the gene in maize was transferred into tobacco leaves. Compared with wild tobacco, the anthocyanin content in juvenile leaves in *Lc*-transgenic lines strains increased at low temperature, showing a purple phenotype. Transcriptome analysis found that *NtDFR* was only significantly expressed in *Lc*-transgenic lines, while the expression of two anthocyanin synthesis related genes *NtAN2* and *NtANS* increased significantly, indicating that *NtDFR* depends on the regulation of *Lc*; again, both *NtAN2* and *Lc* can regulate the expression of *NtANS*, thereby regulating the synthesis of anthocyanins (Huang et al., 2012). This is similar to our research results, which showed the two non-synonymous mutation sites (SNP 7:26887726 and SNP 5:43550809) are highly correlated with cyanidin 3-*O*-glucoside content in the coding regions of *Si012401m* and *Si000845m*. They may serve as sites in response to low-temperature induction and regulate cyanidin 3-*O*-glucoside content at the post-transcriptional level by affecting gene function. What is more, the distribution of allele frequencies that can increase cyanidin 3-*O*-glucoside content in millet in India, the south and north of China gradually increases, which is contrary to the temperature changes in these regions.

Luteolin 7-*O*-glucoside and kaempferol 3-*O*-glucoside as typical representatives in flavonoid aglycones identified five glycosyltransferase genes *Si024831m*, *Si021855m*, *Si021892m*, *Si025159m*, and *Si025028m* within a 200 kb range (14.356–14.376 Mb) on chromosome 3, which are homologous to the three glycosyltransferases *OsUGT705A1*, *OsUGT705A2*, and *OsUGT705A3* in rice, and these three genes in rice are also located in a small interval and have been confirmed to have flavonoid 7-*O*-glycosyltransferase and flavonoid 3′-*O*-glycosyltransferase activity (Peng et al., 2017). So *Si024831m*, *Si021855m*, *Si021892m*, *Si025159m*, and *Si025028m* in millet may also have the function of transferring glycosyl groups to flavonoids. Correspondingly, the frequency of alleles that increase the content of luteolin 7-*O*-glucoside and kaempferol 3-*O*-glucoside is significantly higher in millet in the north of China than in India. The increase in flavonoid aglycones content such as luteolin 7-*O*-glucoside and kaempferol 3-*O*-glucoside helps plants withstand low temperatures environment. Metabolomics-based genome-wide association analysis (mGWAS) has been widely used in Arabidopsis, rice, and maize, deepening our cognition of the genetic basis of these crop metabolomes, greatly promoting the development of functional genomics. Our research fills the gap in the application of mGWAS in millet and paves the way for future dissection of complex physiological traits in millet.

## Conclusion

In the present study, LC-MS-based widely targeted metabolic profiling analysis was performed in a wide range of millet accessions. We observed differentially accumulated patterns of metabolites, including flavonoids and phenolamides, between the millets from India and the south and north of China. Moreover, we researched the genetic basis of metabolites by mGWAS analysis found that cyanidin 3-*O*-glucoside and quercetin *O*-acetylhexside are concentratedly located at 43.55 Mb on chromosome 5 and 26.9 Mb on chromosome 7, and two *Lc* were mined as candidate genes, respectively. However, the signals of luteolin 7-*O*-glucoside and kaempferol 3-*O*-glucoside were also detected at 14.36 Mb on chromosome 3, and five glycosyltransferase genes on this loci were deemed to regulate their content. Then, we used phylogenetic analysis of candidate genes and reported genes to further determine their functions. Our work is the first research to use mGWAS in millet, and it paves the way for future dissection of complex physiological traits in millet.

## Data Availability Statement

The data presented in the study are deposited in the European Variation Archive (EVA) repository, accession number PRJEB45075.

## Author Contributions

ZZ and XW designed the research. WW and SL supervised this study. WW, XZ, GSh, GSo, WYZ, JP, DW, XL, YZ, TT, and CX participated in the material preparation. JS, WW, FZ, XW, FW, XF, and HL carried out the metabolite analyses. YW, QZ, BW, SL, WQZ, and GF performed the annotation of the metabolites and performed the data analysis. WW and SL discussed the results and wrote the manuscript. All authors contributed to the article and approved the submitted version.

## Conflict of Interest

YW, BW, QZ, CX, TT, JS, WQZ, and HL were employed by the company Wuhan Metware Biotechnology Co., Ltd. The remaining authors declare that the research was conducted in the absence of any commercial or financial relationships that could be construed as a potential conflict of interest.

## Publisher’s Note

All claims expressed in this article are solely those of the authors and do not necessarily represent those of their affiliated organizations, or those of the publisher, the editors and the reviewers. Any product that may be evaluated in this article, or claim that may be made by its manufacturer, is not guaranteed or endorsed by the publisher.

## Footnotes

1
http://www.merck-chemicals.com

2
http://www.anpel.com.cn/cnw

3
http://www.biobiopha.com

4
http://www.sigmaaldrich.com

5
http://www.anpel.com.cn/

6
www.shimadzu.com.cn/

7
www.appliedbiosystems.com.cn/

8
http://cran.r-project.org/web/packages/gplots/index.html

9
http://www.ncbi.nlm.nih.gov/
