# Supplementary material for: Metabolome-Based Genome-Wide Association Study Provides Genetic Insights Into the Natural Variation of Foxtail Millet
Source: Front Plant Sci. 2021 Jul 27;12:665530. doi: 10.3389/fpls.2021.665530 (PMC8353534; doi:10.3389/fpls.2021.665530)
Supplement: Supplementary file 1 [file Data_Sheet_1.pdf]

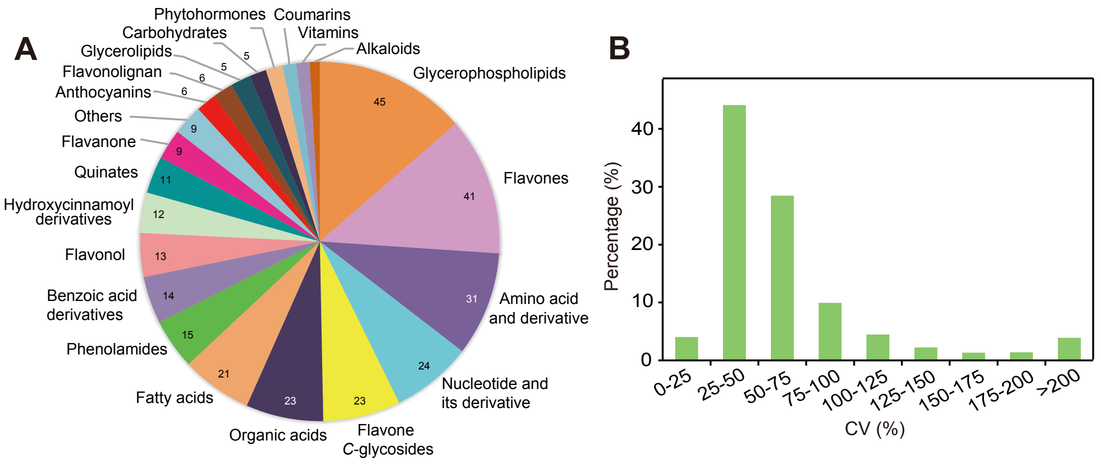

Figure S1. Metabolic profiling in the leaf of foxtail millet. **(A)** Classification of 330 identified/annotated metabolites in millet leaf. **(B)** Distribution of the genetic coefficients of variation (CV) of 1335 metabolic traits in the foxtail millet population.

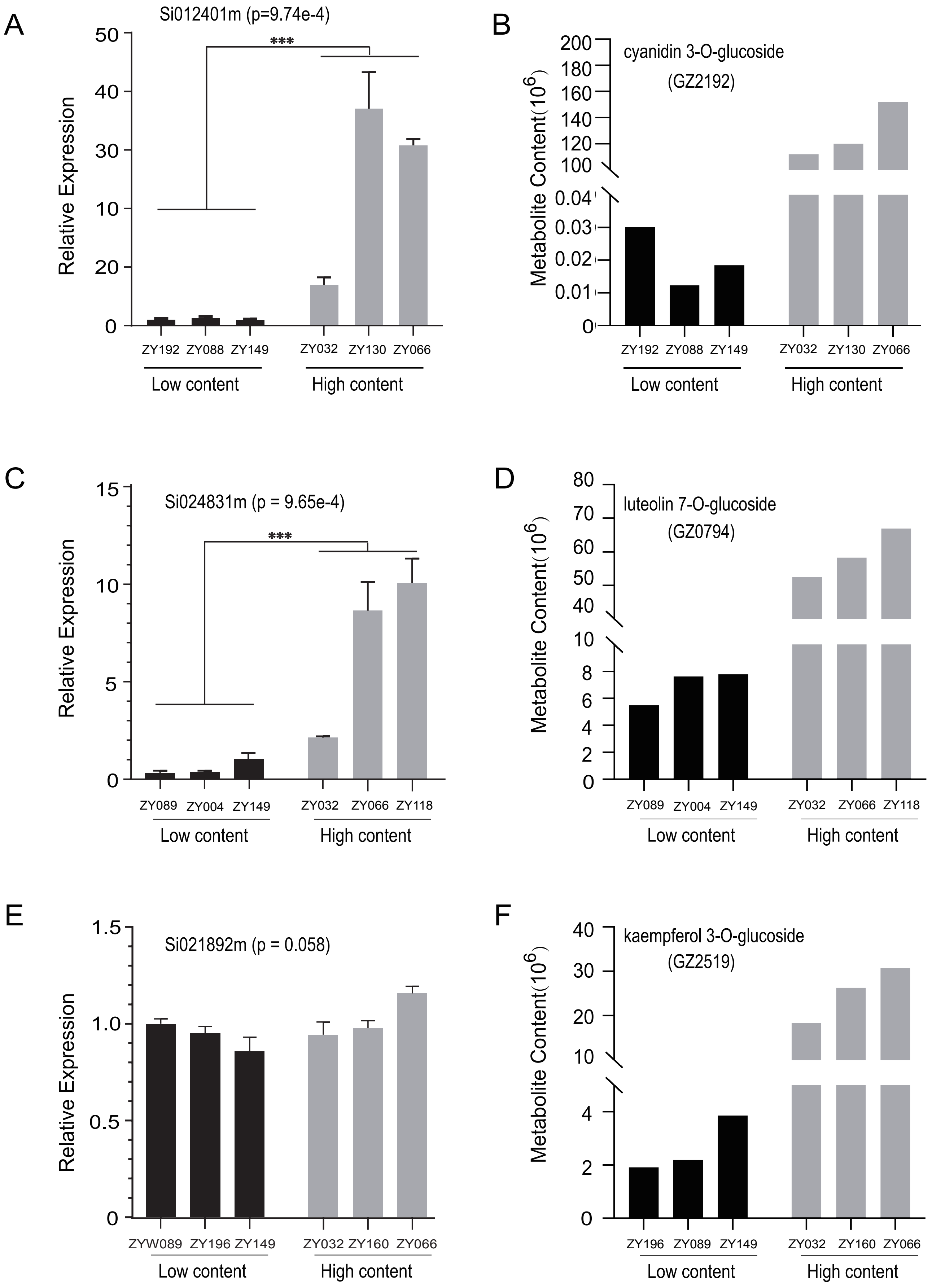

Figure S2. Candidate gene expression profiling and the accumulation of regulated metabolites in millet leaves of different genotypes. **(A-B)** The expression level of Si012401m **(A)** and the content of cyanidin 3-O-glucoside (GZ2192) **(B)** in different genotype materials. **(C-D)** The expression level of Si024831m **(C)** and the content of luteolin 7-O-glucoside (GZ0794) **(D)** in different genotype materials. **(E-F)** The expression level of Si021892m **(E)** and the content of keampferol 3-O-glucoside (GZ2519) **(F)** in different genotype materials. Data represent mean based on three biological replicates, Error bar indicated s.d. based on three biological replicates.(*** $p < 0.001$, Student's *t*-test)
